# Molecular Detection of *Sarcocystis suihominis* and *Sarcocystis miescheriana* in Wild Boars Hunted for Human Consumption in Poland Highlights a Potential Risk for the Consumer

**DOI:** 10.1111/zph.70092

**Published:** 2026-09-07

**Authors:** Weronika Korpysa‐Dzirba, Selene Rubiola, Ewa Bilska‐Zając, Francesco Chiesa, Mirosław Różycki, Aneta Bełcik, Małgorzata Samorek‐Pieróg, Stella Lovisolo, Petras Prakas, Agnė Baranauskaitė, Jacek Karamon, Jacek Sroka, Tomasz Cencek

**Affiliations:** ^1^ Department of Parasitology and Invasive Diseases National Veterinary Research Institute Puławy Poland; ^2^ Department of Veterinary Sciences University of Turin Torino Italy; ^3^ Department of Preclinical Sciences and Infectious Diseases, Faculty of Veterinary Medicine and Animal Science Poznań University of Life Sciences Poznań Poland; ^4^ Regione Piemonte Assessorato alla Sanità, Direzione Sanità‐Settore “Prevenzione, Sanità Pubblica, Veterinaria e Sicurezza Alimentare” Torino Italy; ^5^ Nature Research Centre Vilnius Lithuania; ^6^ Department of Health Biohazards and Parasitology Institute of Rural Health Lublin Poland

**Keywords:** food safety, *Sarcocystis miescheriana*, *Sarcocystis suihominis*, wild boar meat, zoonotic sarcocystosis

## Abstract

**Introduction:**

Wild boars and domestic pigs can act as intermediate hosts for two *Sarcocystis* species: *Sarcocystis miescheriana* and the zoonotic *Sarcocystis suihominis*. Humans become infected with *S. suihominis* by eating raw or undercooked meat. Herein we investigated the occurrence of *Sarcocystis* spp. in wild boars hunted for human consumption in Poland.

**Methods:**

Diaphragms were collected from 241 wild boars during two hunting seasons; a multiplex‐PCR protocol targeting the mtDNA *cox1* gene of *S. miescheriana* and *S. suihominis* was applied; diaphragm samples testing positive for *S. suihominis* underwent sarcocysts isolation and molecular characterization.

**Results:**

Out of 241 wild boars, 216 (90%) revealed the presence of *S. miescheriana* DNA, including eight (3.3%) co‐infected with *S. suihominis*. The diaphragms of three wild boars testing positive for *S. suihominis* and *S. miescheriana* were further processed, yielding 37 sarcocysts. Two out of 37 isolated sarcocysts tested positive for *S. suihominis* and were further molecularly characterized, showing 98.9%–100% identity on the 18S rRNA gene and 98.7% identity on the *cox1* mtDNA gene with *S. suihominis*. Thirteen of the remaining sarcocysts were molecularly characterized, leading to the identification of *S. miescheriana* in 12 cysts; one out of the 13 sarcocysts yielded a 1053 bp *cox1* mtDNA sequence and a 923 bp 18S rDNA sequence showing < 89.1% and 95.6% homology, respectively, with any known *Sarcocystis* species.

**Conclusions:**

This is the first study identifying *Sarcocystis* spp. circulating in wild boars intended for human consumption in Poland, revealing a non‐negligible rate of infection with the zoonotic *S. suihominis,* a high rate of infection with *S. miescheriana* and the presence of a putative new *Sarcocystis* sp. Our study highlights that the risk for the consumers of undercooked wild boar meat cannot be excluded.

## Impacts

- Wild boars hunted for human consumption in Poland were tested for *Sarcocystis* spp., revealing the presence of the zoonotic *Sarcocystis suihominis* in 3.3% of samples.
- The canid‐transmitted *Sarcocystis miescheriana* was detected in 90% of wild boars; concurrently, a putative new *Sarcocystis* sp. using wild boar as intermediate hosts was identified.
- Humans consuming raw or undercooked wild boar meat infected by *Sarcocystis suihominis* can develop sarcocystosis; our results highlight that the risk for the consumers cannot be excluded.

## 1 Introduction

During the last decades, the consumption of wild game meat has increased in European countries, mainly due to the consumer demand for a more ‘natural’, ethical and sustainable meat. Despite this increasing trend, game meat consumption in Europe is still quite low, with only 2%–4% of the population reporting regular consumption, mainly limited to hunters and their relatives and friends (Soriano and Sánchez‐García 2021). Among the reasons behind these data, food safety concerns of the consumer towards wild game play an important role. Nevertheless, hunting of game animals remains an important activity in many European countries; in this context, wild boars ( *Sus scrofa* ) are among the most hunted wild game in Europe and the most hunted game animals in Poland (Rutkowska‐Mazur et al. 2023; Czarniecka‐Skubina et al. 2022).

*Sarcocystis* is a genus of Apicomplexan parasites comprising more than 200 species with a dixenous life cycle, requiring a predator (definitive host) and a prey (intermediate host). Wild boars and domestic pigs can serve as intermediate hosts for at least two *Sarcocystis* species: *Sarcocystis miescheriana* and *Sarcocystis suihominis*. Dogs, foxes, wolves, jackals, raccoon dogs (Canidae) and probably raccoons (Procyonidae) (Odening 1998) can act as definitive hosts for *S. miescheriana*, while humans serve as definitive hosts for *S. suihominis* (Dubey et al. 2016). According to data from the 1970s, a third species of *Sarcocystis* infecting pigs was detected in the former Sovietic Union and named *Sarcocystis porcifelis* (Gazzonis et al. 2019). However, the existence of this species has not been confirmed.

Wild boars and domestic pigs can be infected by *Sarcocystis* spp. after ingesting sporulated oocysts or free sporocysts released by the definitive hosts through the faeces (Coelho et al. 2015). Both *S. suihominis* and *S. miescheriana* can be pathogenic for suids; nevertheless, symptoms depend on the number of ingested oocysts and on the overall health condition of the animals. Although boars' sarcocystosis typically leads to subclinical infections, experimental studies have proven that the consumption of more than one million sporocysts can cause weight loss, thrombocytopenia, skin purpura, muscle tremors, shortness of breath, miscarriage, and even death in pigs (Daugschies et al. 1987).

Only one case of clinical sarcocystosis in pigs following natural infection has been reported, with a fatal outcome due to severe myocarditis (Caspari et al. 2011). Although no further clinical cases have been described, studies conducted in Europe reported a prevalence of *Sarcocystis* spp. infection in domestic pigs ranging from 3% to 43% (Damriyasa et al. 2004; Dubey et al. 2016). However, most of these investigations were performed many years ago in pigs raised under intensive production systems and did not differentiate *S. miescheriana* from *S. suihominis*. More recent studies confirmed the occurrence of *S. suihominis* in domestic pigs in Japan, Spain, China, India and Brazil (Chen et al. 2007; González et al. 2006; Kaur et al. 2016; Saito et al. 1995; Huang et al. 2019; da Rosa et al. 2024).

In recent years, research has increasingly focused on wild boars, in which *Sarcocystis* spp. infection is consistently reported at higher prevalence than in domestic pigs, generally ranging from 61.2% to 97% across Europe and South America (Coelho et al. 2015; Calero‐Bernal et al. 2016; Gazzonis et al. 2019; Moré et al. 2025; Prakas, Kirillova, et al. 2020; Helman et al. 2023; Dimzas et al. 2024; da Rosa et al. 2024). Most studies reported a medium to high prevalence of *S. miescheriana*, and a very low or absent occurrence of *S. suihominis*, which was detected in 1% of wild boars in Italy, in a wild boar in Spain, in 3.6% of wild boars in Argentina, in 1.75% of wild boars in Switzerland and in 3.8% of wild boars in Brazil (Calero‐Bernal et al. 2016; Gazzonis et al. 2019; Helman et al. 2023; Moré et al. 2025; da Rosa et al. 2024). Routine meat inspection, based on visual examination and artificial digestion for the detection of *Trichinella* spp. larvae, does not usually allow to detect sarcocysts. Nevertheless, incidental reports of macroscopic sarcocystosis in pigs and wild boars at slaughter have been recently recorded in Italy, Nigeria, Grenada (Central America), Portugal and Poland (Rubiola et al. 2023; Obadiah et al. 2024; Chikweto et al. 2024; Vieira‐Pinto et al. 2024; Korpysa‐Dzirba et al. 2024).

As previously mentioned, *S. suihominis* may pose a risk for consumers of raw or undercooked pork meat (Pacifico et al. 2023), leading to human intestinal sarcocystosis. This disease is usually asymptomatic or mild‐symptomatic, with gastrointestinal symptoms such as abdominal pain, diarrhoea, nausea, and fever arising within 3 h after the consumption of meat containing sarcocysts and lasting up to 36 h (Rosenthal 2021); the symptom intensity is proportional to the amount of consumed meat (Fayer 2004). According to the data collected by Official Veterinarians in Poland, between 2015 and 2023, a total of 98 cases of swine sarcocystosis have been recorded during post‐mortem examinations in slaughterhouses (www.wetgiw.gov.pl); no data are available for wild boars. Sarcocystosis is not notifiable within the European Union (EU), and no specific regulations concerning the handling of meat containing sarcocysts are available. Therefore, Commission Implementing Regulation (EU) 2019/627, stating that meat intended for human consumption cannot contain parasites, must be applied. As most practitioners do not consider parasites in their initial diagnoses as a possible source of gastrointestinal diseases, the real incidence of human sarcocystosis is unknown, and many infections may go unreported (Nimri 2014). The lack of actual data on the prevalence of *Sarcocystis* spp. in animals and foodstuffs has been highlighted by the European Food Safety Authority (EFSA) in a scientific report published in 2010, emphasizing the need for harmonized schemes for the monitoring and reporting of *Sarcocystis* spp. in food products and in meat‐producing animals (Taylor et al. 2010). Based on the mentioned scientific report, the prevalence of human sarcocystosis in Europe should range between 1.6% and 10.4%; however, these numbers are dated and based only on the microscopic analysis of faecal samples. According to the recent revision of Regulation (EU) 2019/627, there is a need for enforced food chain information and surveillance strategies to deal with the new risk‐based meat inspection strategies. Monitoring emerging foodborne zoonoses is especially important in the present context of growing demand for organic, traditional food products, including traditional homemade meat products such as sausages made of raw or minimally heat‐treated pork originating from home slaughtered backyard pigs or hunted wild boars (Imre et al. 2017). In Poland, the consumption of raw meat is claimed to be approximately 0.5 kg per person per year; however, some groups of people, such as hunters and their families, consume greater amounts of game meat. Moreover, different provinces show different dietary habits, and in some voivodeships there is a strong tradition of consuming food products made from raw or undercooked meat (Rutkowska‐Mazur et al. 2023; Czarniecka‐Skubina et al. 2022). Therefore, further investigation and monitoring are needed to better understand and assess the potential risk of contracting foodborne parasites, such as *S. suihominis*, through the consumption of wild boar meat, in order to safeguard public health and ensure food safety.

The aim of the present study was to determine the occurrence of *S. miescheriana* and *S. suihominis* in wild boars hunted for human consumption in Poland, in order to evaluate the risk for the consumers of wild boar meat.

## 2 Materials and Methods

### 2.1 Sample Collection and DNA Extraction

During the 2022–2023 and 2023–2024 hunting seasons, the diaphragmatic muscle samples of 125 and 116 wild boars hunted for human consumption in the 16 Polish Provinces (Lower Silesian, Kuyavian‐Pomeranian, Lublin, Lubusz, Łódź, Lesser Poland, Masovian, Opole, Subcarpathian, Podlaskie, Pomeranian, Silesian, Świętokrzyskie, Warmian‐Masurian, Greater Poland and West Pomeranian) were collected as part of the routine official inspection for *Trichinella* spp. at the National Veterinary Research Institute (NVRI) in Pulawy (Poland). Following completion of the official testing, the remaining tissue samples (*n* = 241) were used for the present study. The sample size was determined by the availability of these samples; therefore, no a priori sample size calculation was performed, and the study was based on a convenience sample. Upon arrival, 10 g of diaphragmatic muscle samples of each wild boar were individually placed in a 50 mL tube and minced with up to 50 mL of phosphate‐buffered saline (PBS) using a tissue homogenizer; the resulting homogenate was filtered with a sieve and centrifuged for 5 min at 600×*g* (Moré et al. 2013). The supernatant was discarded, and 1 mL of pellet was submitted to DNA extraction using the DNeasy Blood and Tissue Kit (QIAGEN, Hilden, Germany), following the tissue protocol. The resulting samples were stored at −20°C for further molecular analysis. The remaining muscle tissue was stored at −20°C when available.

### 2.2 Molecular Detection of Sarcocystis Suihominis and Sarcocystis Miescheriana by Multiplex‐PCR

The DNA samples were processed applying the multiplex‐PCR protocol targeting the cytochrome C oxidase subunit I mitochondrial gene (mtDNA *cox1*) described by Pacifico et al. (2023) to detect and discriminate *S. suihominis* and *S. miescheriana* using a single forward primer (Cox1 SM) and two reverse primers (Cox1 S and Cox1 M) (Pacifico et al. 2023). DNA extracted from wild boar muscle samples previously confirmed to be negative for *Sarcocystis* spp. by PCR, together with a no‐template control (PCR‐grade water), was included as negative controls in each PCR run. DNA from *S. miescheriana* and *S. suihominis* previously identified in wild boar muscle tissue from Italy (Gazzonis et al. 2019) was included as positive controls. PCR products were separated in 2% agarose gel stained with SYBR Gold Nucleic Acid Gel Stain (Invitrogen by ThermoFisher Scientific, USA) and observed in a UV transilluminator (GelDoc XR Imaging System, BioRad, Hercules, CA, USA). Samples showing the ∼140 bp and/or ∼400 bp amplicons were considered positive for *S. miescheriana* and/or *S. suihominis*, respectively. PCR products corresponding to *S. suihominis* (∼400 bp amplicons) were purified through the ExoSAP‐IT Express treatment (Thermo Fisher Scientific, USA) and sequenced using the BigDye Terminator v3.1 Cycle Sequencing Kit (Applied Biosystems by Thermo Fisher Scientific, Foster City, CA). Sequenced fragments were purified using the NucleoSEQ kit for dye terminator removal (Macherey‐Nagel, Germany) and run on an Applied Biosystems SeqStudio Genetic Analyser (ThermoFisher Scientific, USA). The generated sequences were analysed using the BLASTn sequence similarity search algorithm on the NCBI NT database (Altschul et al. 1990). Differences in prevalence between hunting seasons and among Provinces were assessed using the chi‐square (*χ* ^2^) test and Fisher's exact test. Statistical significance was set at *p* < 0.05. Only confirmed cases of *S. suihominis* were included in the statistical analysis.

### 2.3 Microscopic Detection and Molecular Characterization of *Sarcocystis* spp. Sarcocysts

Muscle samples that were positive for the presence of *S. suihominis* were recovered in order to proceed with the isolation and molecular characterization of single sarcocysts. Up to 10 g of diaphragmatic muscle samples were placed in a 50 mL tube and minced with sterile PBS to a volume of 50 mL using a tissue homogenizer, applying the protocol described by Moré et al. (2013) with slight modifications. The resulting homogenate was filtered using a sieve and centrifuged at 600×*g* for 5 min. The supernatant was discarded, the pellet was resuspended in 20 mL of PBS and observed using an inverted microscope at 40× magnification (Nikon TMS). The detected sarcocysts were individually collected and submitted to DNA extraction using the DNeasy Blood and Tissue Kit (QIAGEN, Hilden, Germany). The resulting single‐sarcocyst DNA samples were molecularly tested with primers Cox1 SM and Cox1 S to identify the *S. suihominis* sarcocysts (Pacifico et al. 2023). Samples resulting positive to *S. suihominis* were molecularly characterized by amplifying and sequencing the partial mtDNA *cox1* gene with the primer pair SF1 and SR11 targeting a fragment of ∼1100 bp as described by Gazzonis et al. (2019), and the complete 18S ribosomal RNA gene (rRNA) using the two primer sets ERIB1—S2 and S3—Primer BSarc (Barta et al. 1997; Fischer and Odening 1998; Gazzonis et al. 2019; Gjerde 2014). A subset of the remaining single‐sarcocyst DNA samples which did not give positive results for *S. suihominis* was molecularly characterized by amplifying and sequencing the partial mtDNA *cox1* gene with primers SF1 and SR11 (Gazzonis et al. 2019). A further molecular characterization was performed on the 18S rRNA gene of a single‐sarcocyst DNA sample whose amplification and sequencing of the mtDNA *cox1* gene revealed the presence of an unidentified *Sarcocystis* sp. After multiple attempts, a newly designed primers set (primer SUNIF1 ACCTGGTTGATCCTGCCAGT and primer SUNIR1 TTCGCAGTAGTTCGTCTTTAACA) was used to amplify and sequence a ∼950–1000 bp fragment of the 18S rRNA gene following a procedure previously described by Prakas, Strazdaitė‐Žielienė, et al. (2020). The software Primer3Plus (Untergasser et al. 2012) was used to create the new primers set. Generated sequences were analysed using MEGA11 (Kumar et al. 2016). Briefly, forward and reverse sequences were assembled into consensus sequences, as well as the 18S rRNA gene overlapping sequences. Nucleotide sequences were trimmed to remove primers and were analysed with the BLASTn sequence similarity search algorithm on the NCBI NT database. The 18S rRNA gene sequences and the mtDNA *cox1* gene sequences obtained were analysed separately, using 59 sequences from 57 taxa for the 18S rRNA gene, including one new sequence generated in this study, and 74 partial sequences from 57 taxa for the mtDNA *cox1* gene, including 14 new sequences generated in this study. The ClustalW algorithm was used to get multiple alignments of the generated sequences and of the sequences retrieved from GenBank. Sequences were truncated at both ends, so that all sequences started and ended at the same nucleotide positions. The final alignments of the mtDNA *cox1* gene and of the 18S rDNA gene comprised 969 and 1904 nucleotide positions, respectively, gaps included. Shorter sequences generated in the present study were removed from the phylogenetic analysis. The phylogenetic trees were reconstructed using the Neighbour‐Joining method, while the evolutionary distances were computed using the Kimura two‐parameter method within MEGA11. In both analyses, the phylogenetic trees were rooted on a sequence of *Toxoplasma gondii*. The phylogeny was tested using the bootstrap test (1000 bootstrap replicates). Accession numbers of the sequences included in the phylogenetic analyses are shown near the species names corresponding to the different branches.

## 3 Results

The application of the multiplex‐PCR protocol revealed the presence of *S. miescheriana* DNA in 217 out of 241 wild boars (217/241, 90%, 95% CI 85.6%–93.3%), eight of which tested positive also for *S. suihominis* (8/241, 3.3%, 95% CI 1.6%–6.5%). No statistically significant differences in the prevalence of *S. suihominis* were observed between hunting seasons (*χ* ^2^ test, *p* = 0.33; Fisher's exact test, *p* = 0.28) or among Provinces (*χ* ^2^ test, *p* = 0.90). The *Sarcocystis* spp. infection rates according to hunting seasons and hunting Provinces are reported in Table S1. The *S. suihominis* positive samples originated wild boars hunted in four Provinces located in the Western and in the Central part of Poland (Zachodniopomorskie, Wielkopolskie, Pomorskie and Łódzkie voivodeships), while *S. miescheriana* was detected in each targeted Province (Figure 1). Sanger sequencing of the eight amplicons resulted in 374–403 bp fragments showing a percentage of identity of 97.3%–99% with *S. suihominis* GenBank entries MH404228, PV769072‐76 and < 82% identity with other *Sarcocystis* spp. sequences available in GenBank.

**FIGURE 1:**
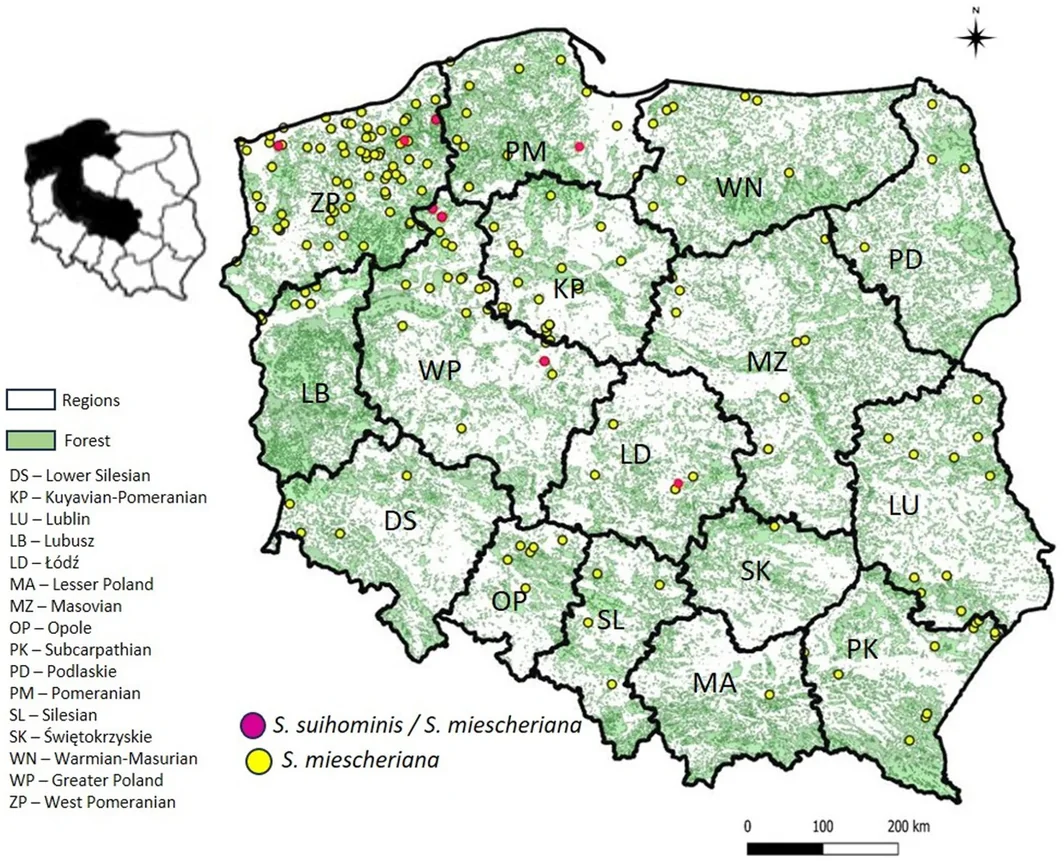
Location of *Sarcocystis* spp. positive wild boars; yellow dot—presence of wild boars affected by *S. miescheriana*; pink dot—presence of wild boars affected by *S. miescheriana* and *S. suihominis*.

The diaphragmatic muscle samples of three out of eight wild boars testing positive for *S. suihominis* were enough to be recovered and processed for the sarcocysts isolation; muscle tissues of the other wild boars could not be recovered. The direct microscopic examination of the muscle homogenates obtained revealed the presence of six, 15 and 16 *Sarcocystis* spp. sarcocysts or cyst‐portions, respectively, in the three diaphragms examined. Out of a total of 37 sarcocysts, two cyst‐portions collected from two different wild boars tested positive for *S. suihominis* and were further molecularly characterized. The partial mtDNA *cox1* gene fragment (∼1100 bp) and the complete 18S rDNA gene of one of the two *S. suihominis* sarcocyst‐portions was successfully amplified, while for the second *S. suihominis* cyst only a ∼400 bp fragment of the mtDNA *cox1* gene (primer pair: Cox1SM and Cox1 S) and a ∼1100 bp fragment of the 18S rDNA gene (primer pair: ERIB1 and S2) could be successfully amplified. Sanger sequencing of the mtDNA *cox1* and of the 18S rDNA amplicons of the first sarcocyst resulted in a 1049 bp and a 1840 bp sequences with a percentage of identity of 98.7% and 98.9%–100%, respectively, with *S. suihominis* GenBank entry MH404228 and with *S. suihominis* GenBank entries MH404229, MK867455, KP732433‐KP732435, OR120386 and < 80.8% and 97.5% identity with *S. miescheriana* or other *Sarcocystis* spp. sequences available in GenBank, respectively. Sanger sequencing of the mtDNA *cox1* and of the 18S rDNA amplicons of the second sarcocyst‐portion resulted in a 376 bp mtDNA *cox1* and a 602 bp 18S rDNA sequences with a percentage of identity of 97.3% and 99.3%–100%, respectively, with *S. suihominis* GenBank entry MH404228 and with *S. suihominis* GenBank entries MH404229, MK867455, KP732433‐ KP732435, OR120386 and < 81.5% and 97.6% identity with *S. miescheriana* or other *Sarcocystis* spp. sequences available in GenBank, respectively. The 376 and 602 bp sequences were excluded from the phylogenetic analyses due to the short length.

The partial mtDNA *cox1* gene fragment (∼1100 bp) of 13 sarcocysts which did not give positive results for *S. suihominis* was successfully amplified and sequenced, yielding twelve 1015–1055 bp sequences with a percentage of identity of 94.1%–99.9% with *S. miescheriana* GenBank entries (accession n. MK867462‐MK867464, LC349977‐LC349980, MH404185‐MH404227, MT070614‐MT070635, OP681505‐OP681509, ON211345‐ON211346), and < 79.9% identity with *S. suihominis* or other *Sarcocystis* spp. sequences available in GenBank. Furthermore, one out of the 13 sarcocysts or cyst‐portions isolated from one of the wild boars concurrently positive for *S. suihominis* and *S. miescheriana* yielded a 1053 bp sequence showing less than 89.1% sequence homology with any known *Sarcocystis* spp. (best match *Sarcocystis japonica*, accession n. OP617452). Multiple attempts made to further characterize the 18S rDNA gene of the unidentified *Sarcocystis* sp. yielded a 923 bp fragment of the 18S rDNA gene showing < 95.6% identity with any known *Sarcocystis* spp. (best match *S. japonica* , accession n. LC349464, LC349445).

The phylogenetic analysis based on the mtDNA *cox1* sequences showed close relationships between the *S. miescheriana* sequences generated in the present study and the *S. miescheriana* specimens isolated in wild boars from Greece, Italy, Latvia and Lithuania, which were all positioned together in a branch; a higher genetic distance was recorded between the *S. miescheriana* sequences obtained herein and the *S. miescheriana* sequences originating from Japan (Figure 2A). Similarly, the 1049 bp *S. suihominis* mtDNA *cox1* sequences generated in the present study showed a close relationship with a *S. suihominis* specimen isolated in a wild boar in Italy; the other *S. suihominis* sequences publicly available were excluded from the analysis due to the short length. The unidentified *Sarcocystis* sp. sequence was placed within a clade including sequences from *S. japonica* , *S. silva*, *S. truncata* , *S. rangiferi*, *S. tarandi* and *S. elongata* , forming a sister group to another clade including *S. entzerothi*, *S. hominis* , *S. sinensis* , *S. bovini* and *S. bovifelis*.

**FIGURE 2:**
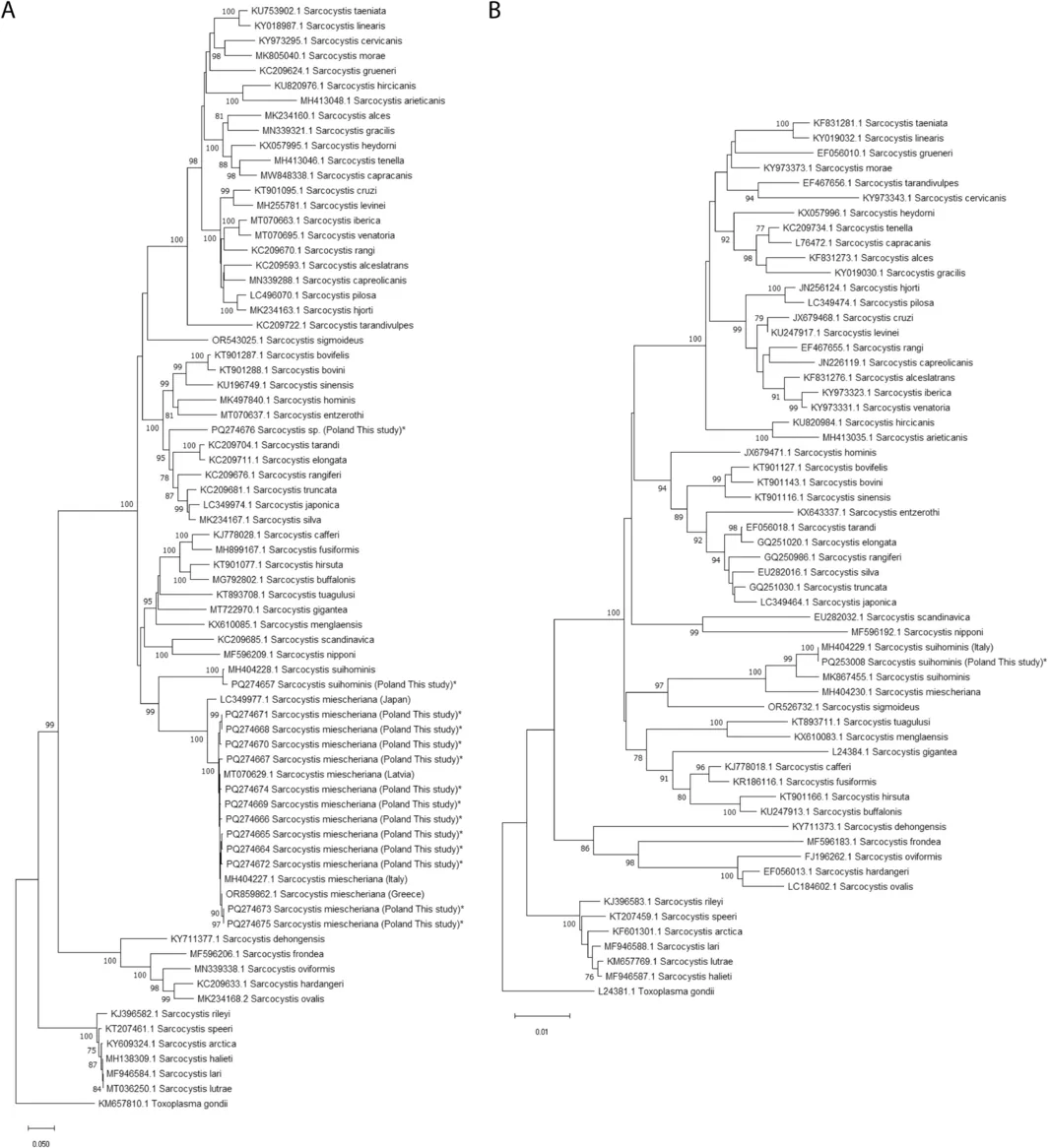
Neighbour‐joining phylogenetic trees based on the alignment of 74 sequences from 57 taxa for the mtDNA cox1 gene (A) and 59 sequences from 57 taxa for the 18S rRNA gene (B), including 15 sequences generated in the present study. Evolutionary distances were computed according to the Kimura two‐parameter method. Branch consensus support is expressed as percentage from 1000 bootstraps and is reported next to the branches. Branch support values lower than 75% are not shown. Trees were rooted on a *Toxoplasma gondii* sequence. GenBank accession numbers are shown next to the branches.

The phylogenetic analysis based on the 18S rDNA sequence showed close relationships between the *S. suihominis* sequence obtained in the present study and the *S. suihominis* specimen isolated in a wild boar in Italy, while a higher genetic distance was recorded between the *S. suihominis* sequence obtained herein and the *S. suihominis* sequences originating from China (Figure 2B). The phylogenetic analysis including the 923 bp 18S rDNA sequence corresponding to the unidentified *Sarcocystis* sp. was conducted separately due to the short length of the fragment (Figure S1), confirming the same phylogenetic position resulted from the mtDNA *cox1* gene analysis.

All the generated mtDNA *cox1* and 18S rDNA sequences were deposited in GenBank under accession numbers PQ274657–PQ274676, PQ253008–PQ253010, PX123326 and PX123327.

## 4 Discussion

In the present study, the occurrence of *S. suihominis* and *S. miescheriana* in wild boars hunted for human consumption in Poland was evaluated, revealing a very high rate of infection with *S. miescheriana* (90%) and a low but non‐negligible rate of infection with *S. suihominis* (3.3%). To the best of the authors knowledge, the occurrence of *Sarcocystis* spp. in wild boars in Poland has only been previously investigated in 2001, showing a low infection rate (∼25%) without distinguishing between the two species (Tropilo et al. 2001); the less sensitive detection method applied by Tropilo et al. (2001), based on the use of microscopy to detect positive samples, might explain the lower prevalence detected. Data on the occurrence of *Sarcocystis* spp. in wild boars in the other European countries ranges from 60.4% (Romania) to 97% (Italy) (Gazzonis et al. 2019; Imre et al. 2017), with various countries reporting prevalence rates of ∼70%–90% (Calero‐Bernal et al. 2016; Coelho et al. 2015; Dimzas et al. 2024; Hvizdosova and Goldová 2009; Pacifico et al. 2023; Prakas 2011; Prakas, Kirillova, et al. 2020; Moré et al. 2025) thereby indicating high level of exposure of wild boars to these protozoa. Nevertheless, as different diagnostic methods were often applied, a comparison between data of different European countries is not always practicable.

While the occurrence of *Sarcocystis* spp. in wild boars appears to be high in most published studies, the literature reporting the application of detection methods allowing to differentiate between *S. miescheriana* and *S. suihominis* reveals a low to very low prevalence of the latter species, which has been detected only in Italy (Gazzonis et al. 2019) and Spain (Calero‐Bernal et al. 2016), with a single positivity detected in both countries, and a reported prevalence of 1% in Switzerland (Moré et al. 2025), in Argentina and in Brazil, where a slightly higher occurrence has been reported (3.6% and 3.8%, respectively) (Helman et al. 2023; da Rosa et al. 2024); in other countries, including Lithuania, Portugal, Romania, USA and Greece, the presence of *S. suihominis* has never been confirmed (Prakas 2011; Coelho et al. 2015; Imre et al. 2017; Calero‐Bernal et al. 2015; Dimzas et al. 2024). Otherwise, higher prevalence rates of *S. suihominis* have been reported in domestic pigs in India and China; these data might be related to the management and slaughtering practices, the poor sanitary conditions and the consumer habits, as previously hypothesised (Helman et al. 2023; Helman et al. 2024; Huang et al. 2019; Kaur et al. 2016; Pacifico et al. 2023). While wild boars should be less exposed to *S. suihominis* oocysts present in infected human faeces with respect to domestic pigs, the higher occurrence of *S. suihominis* reported in the present study highlights a non‐negligible level of environmental contamination by sporocysts of human origin. In areas where the access to sanitation infrastructure is limited or sewage treatment is insufficient, untreated wastewater released into natural water bodies may enhance the environmental circulation of the parasite and increase the risk of exposure for wildlife. Indeed, in rural areas of Poland, access to centralized sewerage systems remains considerably lower than in urban areas, and many households rely on septic tanks or small decentralized wastewater treatment systems, potentially leading to the release of insufficiently treated wastewater into the environment (Piasecki 2019). The traditional consumption of raw or poorly cooked game meat further contributes to maintenance of the parasite's life cycle. Indeed, in Poland, the consumption of traditional food, including meat without or with limited heat treatment, has increased significantly in the last decades, mainly due to the fact that these products are considered to be high quality, made with natural raw materials and sustainable (Szafrańska and Sołowiej 2021). In particular, in the West part of Poland, the tradition of consuming raw meat products is very strong; this habit might explain the detection of *S. suihominis*‐positive wild boars in Wielkopolskie, Zachodniopomorskie, Łódzkie and Pomorskie Provinces, the above‐mentioned Provinces resulting from the present investigation. However, this study was based on a convenience sample consisting of diaphragm tissues remaining after routine official meat inspection. Consequently, the sample was not intended to be representative of each Polish Province. Moreover, the low number of *S. suihominis*‐positive wild boars may have limited the statistical power to detect differences among hunting seasons and Provinces. Therefore, the absence of statistically significant differences, as well as the regional distribution observed in the present study, should be interpreted with caution. Further studies based on larger, geographically stratified sample sizes would be valuable to better investigate the spatial and temporal distribution of *S. suihominis* in Poland.

Although *S. suihominis* is a recognized foodborne zoonotic parasite, epidemiological data in humans remain scarce. Intestinal sarcocystosis is usually asymptomatic or associated with mild, self‐limiting gastrointestinal symptoms, including nausea, abdominal pain and diarrhoea; more severe symptoms have been reported following the ingestion of large numbers of sarcocysts. Experimental human infections have shown that clinical manifestations tend to be more severe following *S. suihominis* infection than *S. hominis* infection (Fayer et al. 2015). Despite its zoonotic importance, the epidemiology of human intestinal sarcocystosis remains poorly understood. Available studies estimated a prevalence ranging from 1.1% to 10.4% in Europe and from 0.4% to 23.2% in Asia. However, these estimates are difficult to compare because study populations, diagnostic methods and species identification differed considerably. Most surveys relied exclusively on light microscopy, and species‐level identification was only rarely achieved, making it impossible to estimate the true occurrence of *S. suihominis* infection in humans (Poulsen and Stensvold 2014). Interestingly, Poland is among the European countries where naturally acquired human intestinal sarcocystosis has previously been reported, although species identification was not available in these historical studies (Fayer et al. 2015). The detection of *S. suihominis* in 3.3% of wild boars hunted for human consumption therefore highlights the need for increased attention to this neglected foodborne parasite, particularly in countries where consumption of raw or undercooked wild boar meat is common. Public education on the importance of thoroughly cooking wild boar meat, together with appropriate sanitation measures to prevent environmental contamination with human faeces, would contribute to reducing transmission of the parasite.

Taking the above into account, the presence of the zoonotic *S. suihominis* in wild boar meat found in this study is the basis for further research to assess the real risk to consumers' health. In line with Regulation (EC) 853/2004, hunters are required to perform an initial inspection of wild game upon capture. Additionally, Regulation (EU) 2017/625 mandates that carcasses infested with parasites must be deemed unsuitable for human consumption. However, the presence of *Sarcocystis* spp. in both wild and domestic swine goes usually unnoticed during meat inspections, due to their microscopic nature. Furthermore, the differentiation of *S. suihominis* and *S. miescheriana* sarcocysts through morphological examination can be difficult and affected by the cyst age and the methods used for fixation (Pacifico et al. 2023). In this context, the application of molecular methods performed herein has allowed the fast and reliable identification of the zoonotic *S. suihominis* in wild boar meat. Moreover, this work contributes to increase the limited number of *S. suihominis* sequences publicly available, which included, so far, only six fragments of mtDNA *cox1* sequences generated from European isolates in wild boars (Gazzonis et al. 2019; Moré et al. 2025), three mtDNA *cox1* sequences generated from Chinese isolates detected in domestic pigs (Huang et al. 2019) and one mtDNA *cox1* sequence generated from a domestic pig in Nigeria (Obadiah et al. 2024), together with around 20 partial 18S rDNA sequences, including only one European isolate (Gazzonis et al. 2019). As the mtDNA *cox1* gene has proven to be a promising tool to differentiate closely related *Sarcocystis* spp. using ungulates as intermediate hosts (Gjerde 2013), and as a high sequence variation between different isolates and different geographical locations can be inferred by the mtDNA *cox1* phylogenetic analysis, the molecular characterization of different *Sarcocystis* spp. isolates should be highly encouraged.

Unlike *S. suihominis*, based on our data and on the data generated in other countries, *S. miescheriana* is widespread in wild boars, reaching up to 90% of occurrence in Poland. This might be explained by the coexistence of the intermediate and definitive hosts, including several wild canids, especially wolves ( *Canis lupus* ) and red foxes ( *Vulpes vulpes* ). Moreover, a role of the hunting dogs in the maintenance of *S. miescheriana* life cycle has been previously hypothesized (Basso et al. 2020; Pacifico et al. 2023). In the present work, all the wild boars testing positive for *S. suihominis* revealed the co‐occurrence of *S. miescheriana* by multiplex‐PCR; moreover, the three wild boars muscle samples subjected to sarcocysts isolation and molecular characterization of the single cysts revealed a higher cyst burden of *S. miescheriana*, as already pointed out by other authors (Helman et al. 2023). The high occurrence of *S. miescheriana* in wild boars, together with the high cyst burden, highlights the possible underreporting of *S. suihominis*, and the resulting need to use molecular and morphological tools enabling a proper detection and differentiation of the two *Sarcocystis* spp. In the present work, the application of the multiplex‐PCR set‐up by Pacifico et al. (2023) and previously applied in Italy (Pacifico et al. 2023) and in Greece (Dimzas et al. 2024) has allowed the fast identification of *S. suihominis* and *S. miescheriana*. Furthermore, the single‐sarcocyst molecular characterization performed herein in three wild boar carcasses co‐infected with *S. miescheriana* and *S. suihominis* revealed the presence of a third, unidentified *Sarcocystis* sp. harboured by wild boars; based on the phylogenetic analyses performed on the mtDNA *cox1* gene (Figure 2), this species was clearly separated from all the other described *Sarcocystis* spp. and was included in a cluster together with other *Sarcocystis* spp. using cervids as intermediate hosts and either confirmed or predicted to use mustelids or felids as definitive hosts, that is *S. tarandi, S. elongata, S. rangiferi, S. japonica*, and *S. silva*. The clade including the putative new *Sarcocystis* sp. was sister to another clade including *Sarcocystis* spp. using bovids as intermediate hosts. The analysis performed on the 923 bp 18S rDNA gene fragment confirmed the same phylogenetic position, which placed the unidentified *Sarcocystis* sp. in a major clade with other *Sarcocystis* spp. using ruminants (Cervidae—*S. entzerothi, S. tarandi, S. elongata, S. silva, S. rangiferi, S. truncata, S. japonica*—and Bovidae—*S. bovifelis, S. bovini, S. sinensis, S. hominis*) as intermediate hosts (Figure S1). A third species of *Sarcocystis* using wild and domestic swine as intermediate hosts was already described before. Indeed, in 1976, in the former Union of Soviet Socialist Republics, Dubey described the presence of a *Sarcocystis* sp. using domestic cats as definitive hosts and pigs as intermediate hosts, and named it *Sarcocystis porcifelis* (Dubey et al. 2016); based on Dubey et al. (2016), sarcocysts of this third cat‐transmitted species have been found in pigs in Romania, in Japan and in India. Nevertheless, the structure of the sarcocysts was not described, and no molecular data have ever been provided, so that *S. porcifelis* is actually considered an invalid *Sarcocystis* species (Dubey et al. 2016). Based on these phylogenetic analyses, mustelids and felids may represent potential definitive hosts. Further research is needed to characterize the putative new species here reported, to investigate its life cycle and to evaluate if this unknown *Sarcocystis* sp. might match with the forgotten *S. porcifelis*.

Although the presence of grossly visible lesions was not recorded in the diaphragmatic samples collected and processed in the present study, the possible association of *S. miescheriana* or *S. suihominis* with macroscopic pathological changes such as cystic lesions has been recently reported in domestic pigs (Chikweto et al. 2024; Korpysa‐Dzirba et al. 2024; Obadiah et al. 2024; Rubiola et al. 2023) and wild boars (Vieira‐Pinto et al. 2024). As the detection of macroscopic parasitic lesions at slaughter leads to carcass condemnation and resulting economic losses, the association of *Sarcocystis* spp. with visible pathological lesions in wild boars and domestic swine should be further investigated.

In conclusion, this is the first study identifying *Sarcocystis* spp. circulating in wild boars hunted for human consumption in Poland, revealing a very high rate of infection with *S. miescheriana*, a non‐negligible rate of infection with the zoonotic *S. suihominis* and the presence of a putative new *Sarcocystis* sp. using boars as intermediate hosts. Although the prevalence of *S. suihominis* detected in this study appears limited, the identification of this zoonotic species in wild boars intended for human consumption deserves attention, particularly considering the traditional consumption of raw or undercooked game meat in some regions. Further research should be conducted to monitor the presence of *S. suihominis* in wild boars and domestic swine meat intended for human consumption in order to evaluate the risk for the consumer of raw or undercooked products.

## Funding

This research did not receive any specific grant from funding agencies in the public, commercial, or not‐for‐profit sectors. Funding for the study was partially provided by the University of Turin, grant ‘Ricerca Locale 2023‐Linea B‐RUBS_RILO_23_01’.

## Ethics Statement

The wild boars used in the present study were legally hunted in accordance with EU legislation, during the hunting seasons and with routine sanitary surveillance. Consequently, ethical approval was not deemed necessary.

## Conflicts of Interest

The authors declare no conflicts of interest.

## Supporting information


**Figure S1:** Neighbour‐joining phylogenetic trees based on the alignment of 60 18S rRNA sequences from 58 taxa, the unidentified *Sarcocystis* sp. sequence generated in the present study (Accession number PQ253010). A total of 948 nucleotide positions were included in the final dataset. Evolutionary distances were computed according to the Kimura two‐parameter method. Branch consensus support is expressed as percentage from 1000 bootstraps and is reported next to the branches. Branch support values lower than 75% are not shown. The tree was rooted on a *Toxoplasma gondii* sequence. GenBank accession numbers are shown next to the branches.

**Table S1:** *Sarcocystis* spp. infection rates according to hunting seasons and hunting Provinces.

## Data Availability

The datasets generated in the present study are available in GenBank database with accession numbers PQ274657‐PQ274676, PQ253008‐PQ253010, PX123326 and PX123327.
