# Supplementary material for: Molecular Detection of Sarcocystis suihominis and Sarcocystis miescheriana in Wild Boars Hunted for Human Consumption in Poland Highlights a Potential Risk for the Consumer
Source: Zoonoses Public Health. 2026 Sep 7;73(8):706–15. doi: 10.1111/zph.70092 (PMC13749141; doi:10.1111/zph.70092)
Supplement: Supplementary file 1 [file ZPH-73-706-s001.docx]

Supplementary figure S1: Neighbour-joining phylogenetic trees based on the alignment of 60 18S rRNA sequences from 58 taxa, the unidentified *Sarcocystis* sp. sequence generated in the present study (Accession number PQ253010). A total of 948 nucleotide positions were included in the final dataset. Evolutionary distances were computed according to the Kimura two-parameter method. Branch consensus support is expressed as percentage from 1000 bootstraps and is reported next to the branches. Branch support values lower than 75% are not shown. The tree was rooted on a *Toxoplasma gondii* sequence. GenBank accession numbers are shown next to the branches.
