# Supplementary material for: Molecular Detection of Sarcocystis suihominis and Sarcocystis miescheriana in Wild Boars Hunted for Human Consumption in Poland Highlights a Potential Risk for the Consumer
Source: Zoonoses Public Health. 2026 Sep 7;73(8):706–15. doi: 10.1111/zph.70092 (PMC13749141; doi:10.1111/zph.70092)
Supplement: Supplementary file 2 [file ZPH-73-706-s002.docx]

| **Variable** | **Category** | **N positive/ N examined** | **Percentage (95% CI)** |
| --- | --- | --- | --- |
| Hunting Province | Dolnośląskie | 3/4 | 75 (19.41-99.37) |
|  | Kujawsko-Pomorskie | 24/26 | 92.31 (74.87-99.05) |
|  | Łódzkie | 6/6 (1/6 *S. suihominis* †) | 100 (54.07-100) |
|  | Lubelskie | 20/22 | 90.91 (70.84-98.88) |
|  | Lubuskie | 11/13 | 84.62 (54.55-98.08) |
|  | Małopolskie | 1/1 | 100 (2.5-100) |
|  | Mazowieckie | 9/9 | 100 (66.37-100) |
|  | Opolskie | 8/8 | 100 (63.06-100) |
|  | Podkarpackie | 3/4 | 75 (19.41-99.37) |
|  | Podlaskie | 4/4 | 100 (39.76-100) |
|  | Pomorskie | 16/16 (1/16 *S. suihominis* †) | 100 (79.41-100) |
|  | Śląskie | 3/3 | 100 (29.24-100) |
|  | Świętokrzyskie | 1/3 | 33.33 (0.84-90.57) |
|  | Warmińsko-Mazurskie | 9/10 | 90 (55.5-99.75) |
|  | Wielkopolskie | 20/21 (3/21 *S. suihominis* †) | 95.24 (76.28-99.88) |
|  | Zachodniopomorskie | 75/86 (3/86 *S. suihominis* †) | 87.21 (78.27-93.44) |
|  | Unidentified location | 4/5 | 80 (28.36-99.49) |
| Hunting season | 2022-2023 | 117/125 (6/125 *S. suihominis* †) | 93.6 (87.78-97.2) |
|  | 2023-2024 | 100/116 (2/116 *S. suihominis* †) | 86.21 (78.57-91.91) |

†co-infection with *S. miescheriana*

Supplementary table S1. *Sarcocystis* spp. infection rates according to hunting seasons and hunting Provinces.
